# Tyr-Trp administration facilitates brain norepinephrine metabolism and ameliorates a short-term memory deficit in a mouse model of Alzheimer’s disease

**DOI:** 10.1371/journal.pone.0232233

**Published:** 2020-05-04

**Authors:** Takashi Ichinose, Hiroyasu Murasawa, Tomoko Ishijima, Shinji Okada, Keiko Abe, Saki Matsumoto, Toshiro Matsui, Shigeki Furuya

**Affiliations:** 1 Department of Innovative Science and Technology for Bio-industry, Graduate School of Bioresource and Bioenvironmental Sciences, Faculty of Agriculture, Kyushu University, Fukuoka, Japan; 2 Hashima Laboratory, Nihon Bioresearch Inc., Hashima, Gifu, Japan; 3 Graduate School of Agricultural and Life Science, The University of Tokyo, Bunkyo-ku, Tokyo, Japan; 4 Group for Food Functionality Assessment, Kanagawa Institute of Industrial Science and Technology, Kawasaki-ku, Kawasaki, Kanagawa, Japan; 5 Innovative Bio-Architecture Center, Faculty of Agriculture, Kyushu University, Fukuoka, Japan; Queen's University Belfast, UNITED KINGDOM

## Abstract

The physiological actions of orally ingested peptides on the brain remain poorly understood. This study examined the effects of 39 orally administered synthetic Tyr-containing dipeptides on the enhancement of brain norepinephrine metabolism in mice by comparing the concentration of 3-methoxy-4-hydroxyphenylethyleneglycol (MHPG). Although Tyr-Tyr administration increased blood and cerebral cortex (Cx) Tyr concentrations the most, Tyr-Trp increased Cx MHPG concentration the most. The oral administration of Tyr-Trp ameliorated a short-term memory deficit of a mouse model of cognitive dysfunction induced by amyloid beta peptide 25–35. Gene expression profiling of mouse brain using a microarray indicated that Tyr-Trp administration led to a wide variety of changes in mRNA levels, including the upregulation of genes encoding molecules involved in catecholamine metabolism. A comparative metabolome analysis of the Cx of mice given Tyr-Trp or Tyr-Tyr demonstrated that Tyr-Trp administration yielded higher concentrations of Trp and kynurenine pathway metabolites than Tyr-Tyr administration, as well as higher L-dopa levels, which is the initial product of catecholamine metabolism. Catecholamines were not significantly increased in the Cx of the Tyr-Tyr group compared with the Tyr-Trp group, despite a marked increase in Tyr. Presumably, Tyr-Trp administration enhances catecholamine synthesis and metabolism via the upregulation of genes involved in Tyr and Trp metabolism as well as metabolites of Tyr and Trp. These findings strongly suggest that orally ingested Tyr-Trp modulates the brain metabolome involved in catecholamine metabolism and contributes to higher brain function.

## Introduction

With increasing life expectancy in Europe, the United States, and Japan, the risk of various aging-associated cognitive and physical dysfunctions is also on the rise. As a result of population aging, the number of patients with dementia has increased rapidly [1]. There are many types of dementia, including vascular dementia and dementia with Lewy bodies, but Alzheimer’s disease (AD) is particularly prevalent. This condition also affects cognitive function as it progresses, reducing not only the quality of life of the patient but also that of their caregiver. There are many hypotheses on the pathogenesis of AD, but its detailed molecular mechanism is unknown, and so no effective preventive or therapeutic measures have been established. The search for AD biomarkers in human AD brain and animal AD models via metabolome analysis using high-sensitivity mass spectrometry (MS) has identified many candidate molecules [2]. Among these candidates, decreased norepinephrine (NE), a key catecholamine neurotransmitter in the brain, has been suspected to be involved in the decline of cognitive function in patients with AD [3]; indeed, NE concentrations are decreased in postmortem AD brains [4,5]. Morphological observations have revealed noradrenergic fiber degeneration in the locus coeruleus of many postmortem AD brains [6,7]. These phenomena are thought to be closely related to the decline in cognitive function observed in patients with AD [8]. It has also been suggested that the onset of AD and abnormal central NE network function are highly correlated [9,10]. Thus, the enhancement of NE synthesis and metabolism as well as the maintenance of central NE function could contribute to the prevention and treatment of AD.

The amino acid tyrosine (Tyr) is a precursor of catecholamines, which are neurotransmitters associated with higher brain functions. Tyr availability in the body influences the synthesis and transmission of catecholamine neurotransmitters in the brain [11,12]. Indeed, the oral intake of Tyr contributes to enhanced cognitive performance under various forms of stress and may also improve mood [13,14]. Blood Tyr crosses the blood-brain barrier via L-type amino acid transporter 1 [15]. However, the metabolic fate of Tyr in proteins and peptides in the brain remains largely unexplored. The oral intake of peptides derived from soy protein increases blood aromatic amino acid concentrations more efficiently than the intake of amino acid mixtures of the same composition [16]. Therefore, the intake of Tyr-containing peptides may result in efficient Tyr absorption and enhanced catecholamine synthesis and metabolism in the brain.

We previously showed that Ser-Tyr dipeptides have high permeability in a human intestinal epithelial cell model and lead to marked increases in 3-methoxy-4-hydroxyphenylethyleneglycol (MHPG) levels and accelerated NE turnover ([MHPG + normetanephrine {NM}] / NE) in the brain after oral intake [17]. MHPG is a metabolite of NE. We compared Ser-Tyr, Ile-Tyr, and Tyr-Pro dipeptides, and found that even if a dipeptide contained Tyr, the degree of its effect on brain NE synthesis and metabolism varied according to the amino acid that Tyr was combined with. Thus, it is plausible that a comprehensive evaluation of Tyr-containing dipeptides might identify peptides that could effectively enhance NE synthesis and metabolism in the brain. Therefore, in the present study, to gain an insight into the regulation of brain NE synthesis and metabolism by Tyr-containing dipeptides, we compared 39 Tyr-containing dipeptides in terms of their MHPG-producing effect in the brain. Tyrosine-tryptophan (Tyr-Trp or YW) was found to be the most effective dipeptide at increasing MHPG concentrations. In addition, we investigated the effect of chronic Tyr-Trp administration on short-term memory in AD model mice. We also conducted DNA microarray analysis using samples from AD model mice that had performed behavioral tests. Furthermore, we performed metabolome analysis to evaluate the Tyr-Trp-specific mechanism for enhanced NE synthesis and metabolism. The present study indicated that the oral administration of Tyr-Trp specifically enhanced brain NE synthesis and metabolism and ameliorated a memory deficit in a mouse model of cognitive dysfunction caused by amyloid beta (Aβ) peptide 25–35.

## Materials and methods

### Ethics approval

All of the animal experiments in this study followed the Standard Relating to the Care and Management of Laboratory Animals and Relief of Pain (Notice No. 88, Ministry of the Environment, Government of Japan). All experiments were reviewed and approved by the Animal Experiment Committee of Kyushu University (Permit No. A19-333) and Nihon Bioresearch, Inc.

### Chemicals and reagents

In total, 39 different Tyr-containing dipeptides for Tyr and NE concentration analysis were purchased from the Peptide Institute, Inc. (Osaka, Japan). Tyr-Trp for animal behavioral tests was synthesized using the 9-fluorenylmethoxycarbonyl-solid phase synthesis method with Respep SL (Intavis Bioanalytical Instruments AG, Köln, Germany). Specially prepared reagent-grade Tyr was purchased from Nacalai Tesque, Inc. (Kyoto, Japan). Distilled water, methanol, acetonitrile, and formic acid, all of liquid chromatography–mass spectrometry (LC-MS) grade, were purchased from Merck Co. (Darmstadt, Germany). All other reagents were analytical grade and used without further purification.

### Animals and treatments for Tyr and NE concentration analysis

Male C57BL/6 mice at 8 weeks of age were purchased from Charles River Laboratories Japan, Inc. (Kanagawa, Japan). They were maintained in a pathogen-free animal facility (25°C ± 1°C) with a 12-h light/12-h dark cycle and provided with CE-2 chow (CLEA Japan, Inc., Tokyo, Japan) and water *ad libitum*.

The mice were divided into 41 groups (*n* = 3 per group) and administered one of the 39 Tyr-containing dipeptides (1 mmol kg^-1^) dissolved in saline, Tyr alone, or saline (Vehicle) alone (20 mL kg^-1^) by oral gavage. Cerebral cortex (Cx) samples were collected at 30 min after oral ingestion and stored at −80°C. Serum samples were prepared by centrifuging blood at 10,000 × *g* for 10 min at 4°C after standing for 1 h at 4°C and then stored at −20°C.

### Animals and treatments for animal behavioral tests

Male ddY mice at 10 weeks of age (SLC, Inc., Shizuoka, Japan) were housed in a room with controlled lighting (12-h light/12-h dark) and constant temperature (25°C) and provided with a moderate-fat diet (Oriental Yeast Co., Ltd., Tokyo, Japan) and water *ad libitum*.

The mice were divided into 3 groups (*n* = 10 per group): Sham group, which received saline administration and distilled water injection; Aβ group, which received saline administration and Aβ peptide 25–35 injection; and Aβ+YW group, which received Tyr-Trp administration and Aβ peptide 25–35 injection. Tyr-Trp was orally administered (100 mg kg^-1^ day^-1^, twice a day), starting 7 days before Aβ peptide injection. The other groups received oral administration of saline (5 mL kg^-1^).

Aβ peptide 25–35 (Peptide Institute, Inc.) was dissolved in distilled water (final concentration 2 mM) and incubated at 37°C for 96 h. Intracerebroventricular injection was performed as described previously with some modification. Briefly, each mouse was anesthetized using an intraperitoneal injection of pentobarbital in saline and a subcutaneous injection of levobupivacaine, and placed in a stereotaxic frame (Narishige, Inc., Tokyo, Japan). A 28-gauge needle was inserted at the following position: 1 mm right of the midline, 0.2 mm posterior, and at a depth of 2.5 mm from the bregma. Aβ peptide 25–35 solution (3 μL, 6 nmol) was injected intracerebroventricularly at a rate of 1 μL/min using a syringe pump. The needle was kept in place for an additional 3 min and then withdrawn.

### Animals and treatments for metabolome analysis

C57BL/6 mice at 8 weeks of age were purchased from Charles River Laboratories Japan, Inc. (Kanagawa, Japan). They were maintained in a pathogen-free animal facility (25°C ± 1°C) with a 12-h light/12-h dark cycle. They were provided with CE-2 chow (CLEA Japan, Inc.) and water *ad libitum*.

The mice were divided into two groups (*n* = 7) and each group was administered Tyr-Trp or Tyr-Tyr (1 mmol kg^-1^) dissolved in saline by oral gavage. The Cx was collected at 30 min after oral ingestion and stored at −80°C.

### Tyr concentration analysis in serum or Cx

The samples were homogenized individually in 5 volumes of water and centrifuged at 20,000 × *g* for 20 min at 4°C. Proteins in the supernatant were removed by adding a 1/10 volume of 60% perchloric acid. The Tyr concentration of the supernatants was determined with high-performance liquid chromatography-MS (Waters Corp., Milford, MA) using an Intrada Amino Acid column (Imtakt, Kyoto, Japan).

### NE and NE metabolites concentration analysis

The Cx was homogenized in 5 volumes of ice-cold 0.2 M perchloric acid containing 0.1 mM EDTA⋅2Na (Dojindo Molecular Technologies, Inc., Kumamoto, Japan) and 10 μL of 10 μg μL^−1^ isoproterenol. The mixture was centrifuged at 20,000 × *g* for 15 min at 4°C. Then, 200 μL of the supernatant was mixed with 35 μL of 0.2 M sodium acetate and filtered through a membrane filter (0.22 μm; Merck Millipore, Burlington, MA). The HTEC-500 system was used for reverse-phase chromatographic analysis (Eicom Co., Ltd., Kyoto, Japan) and consisted of an Eicompack SC-5ODS column (3 mm φ × 150 mm; Eicom Co., Ltd.) and a graphite carbon working electrode (Eicom WE-3G; 12 mm φ) with an Ag/AgCl reference electrode. Electrochemical detection potential was set at +750 mV for the working electrode.

### Behavioral testing

Mouse behavioral testing was performed as described by Kobayashi et al. [18]. A Y-maze (YUNIKOMU Co., Ltd., Gifu, Japan) test was performed at 6 days after intracerebroventricular injection to assess the working memory of the mice. Room light was controlled and maintained between 10–40 lx. The maze consisted of polyvinyl plastic and had three arms (395 mm deep, 120 mm high, 45 mm wide at the bottom and 100 mm wide at the top) at angles of 120°. The mice were placed at the end of one arm and allowed to move freely for 7 min. The sequence of arm entries was counted manually to calculate the total number of entries, and the spontaneous alternation ratio was calculated as follows: (number of spontaneous alterations) / (number of total entries − 2) × 100. The test was performed by a person blinded to group assignment.

### DNA microarray analysis

Total RNA was isolated from each Cx sample using TRIzol (Thermo Fisher Scientific, Waltham, MA) and subsequently purified with an RNeasy Mini Kit (Qiagen, Hilden, Germany) and RNase-Free DNase Set (Qiagen), according to the manufacturers’ protocols. Total RNA quality and quantity were assessed by agarose gel electrophoresis and spectrophotometry.

DNA microarray analysis was performed on Cx samples from a total of 15 mice (*n* = 5 each from the Sham, Aβ, and Aβ+YW groups) by choosing average individuals from each group on the basis of the spontaneous alternation ratio.

For each sample, biotinylated single-stranded cDNA was synthesized from 100 ng total RNA using a GeneChip WT PLUS Reagent Kit (Thermo Fisher Scientific). The cDNAs were subsequently hybridized to a Clariom S Mouse Array (Thermo Fisher Scientific). The arrays were washed and labeled with streptavidin–phycoerythrin using a GeneChip Hybridization, Wash, and Stain Kit and Fluidics Station 450 system (Thermo Fisher Scientific). Fluorescence was detected using a GeneChip Scanner 3000 7G (Thermo Fisher Scientific).

For DNA microarray analysis, Affymetrix GeneChip Command Console software was used to reduce the array images to the intensity of each probe (CEL files). The CEL files were quantified using the Factor Analysis for Robust Microarray Summarization algorithm (quantile normalization, qFARMS) [19] and the Robust Multi-array Average [20] with the statistical packages R [21] and Bioconductor [22]. Probe sets found to be differentially expressed in the Aβ group vs. Sham group or Aβ+YW group vs. Aβ group were identified according to the rank products method [23] for qFARMS-normalized data using R. The fold change values for the probe sets between the groups were calculated using Robust Multi-array Average-normalized data. Because qFARMS principal component analysis showed that one sample (G1.2) in the Sham group was located very far from the other samples (S1 Fig), this sample was omitted from further analysis (S2 Fig). The results of statistical analysis of the spontaneous alternation ratio were not changed by omitting G1.2 (data not shown). Probe sets that showed a false discovery rate <0.05 were considered to be differentially expressed genes (DEGs). Gene-annotation enrichment analysis between the Aβ and Aβ+YW groups was then performed using the web tool Database for Annotation, Visualization, and Integrated Discovery 6.8 (DAVID; http://david.abcc.ncifcrf.gov/) with Kyoto Encyclopedia of Genes and Genomes (KEGG) pathway analysis. KEGG pathways with a Benjamini-Hochberg false discovery rate <0.05 were regarded as significantly enriched according to the developer’s protocol [24].

### Targeted metabolome analysis

Each Cx was homogenized in 5 volumes of ice-cold water and 10 μL of 100 μmol μL^−1^ 2-morpholinoethanesulfonic acid as an internal standard. The mixture was centrifuged at 20,000 × *g* for 5 min at 4°C. The supernatant was filtered with an ultrafiltration filter (Amicon Ultra 0.5 Centrifugal Filter Unit Ultracel-3; Sigma-Aldrich, St. Louis, MO) at 15,000 × *g* for 30 min at 4°C. The filtrate was passed through a solid phase extraction centrifuge column (MonoSpin C18; GL Sciences, Inc., Tokyo, Japan) and passed through a membrane filter (0.22 μm; Merck Millipore).

Metabolome analysis was performed with the Nexera X2 system (Shimadzu Corp., Kyoto, Japan) equipped with two LC-30 AD pumps, a DGU-20A_5R_ degasser, an SIL-30 AC autosampler, a CTO-20 AC column oven, and a CBM-20A control module, coupled with an LCMS-8060 triple quadrupole mass spectrometer (Shimadzu Corp.). A pentafluorophenylpropyl column (Discovery HS F5, 150 × 2.1 mm, 3 μm; Sigma-Aldrich) was used to separate metabolites and was maintained at 40°C. The injection volume was 2 μL. Mobile phase A was 0.1% formic acid water, while mobile phase B was 0.1% formic acid acetonitrile. Targeted metabolites and the internal standard (2-morpholinoethanesulfonic acid) were eluted with a linear gradient starting at 5% mobile phase B and progressing to 100% mobile phase B in 20 min; the conditions were kept at 100% mobile phase B for 5 min, after which the column was equilibrated to the starting conditions from 25 to 30 min. The electrospray ionization source parameters were as follows: interface temperature 300°C, desolvation line temperature 250°C, heating block temperature 400°C, nebulizing gas flow rate 3 L/min, heating gas flow rate 10 L/min, drying gas flow rate 10 L/min, interface voltage for positive mode +4.5 kV, interface voltage for negative mode -3.5 kV, and collision-induced dissociation gas pressure 230 kPa. Targeted metabolites and the internal standard were measured using multiple reaction monitoring in either positive or negative ion mode with the complete optimized target list shown in S1 Table. Peak detection, integration, and quantification were performed with Lab Solutions Analysis Software (Shimadzu Corp.). The relative ratios of metabolite peak areas to the peak areas of the internal standard were used for statistical analyses. When the metabolome data were analyzed, orthogonal projections to latent structure discriminant analysis (OPLS-DA) was carried out and OPLS-DA score plots and loading S-plots were generated using SIMCA software (Umetrics AB, Umea, Sweden).

### Statistical analysis

The results are expressed as the mean ± standard error of the mean (S.E.M.). NE, NE metabolites, and NE turnover were analyzed using one-way analysis of variance followed by Dunnett’s test for *post hoc* analysis. Behavioral testing was analyzed using one-way analysis of variance followed by Tukey’s honestly significant difference test for *post hoc* analysis. All analyses were carried out with SPSS (IBM, Armonk, NY), and *p*-values <0.05 were considered significant.

## Results

### Tyr-Trp administration effectively increases MHPG concentration and NE turnover in the Cx

**Fig 1:**
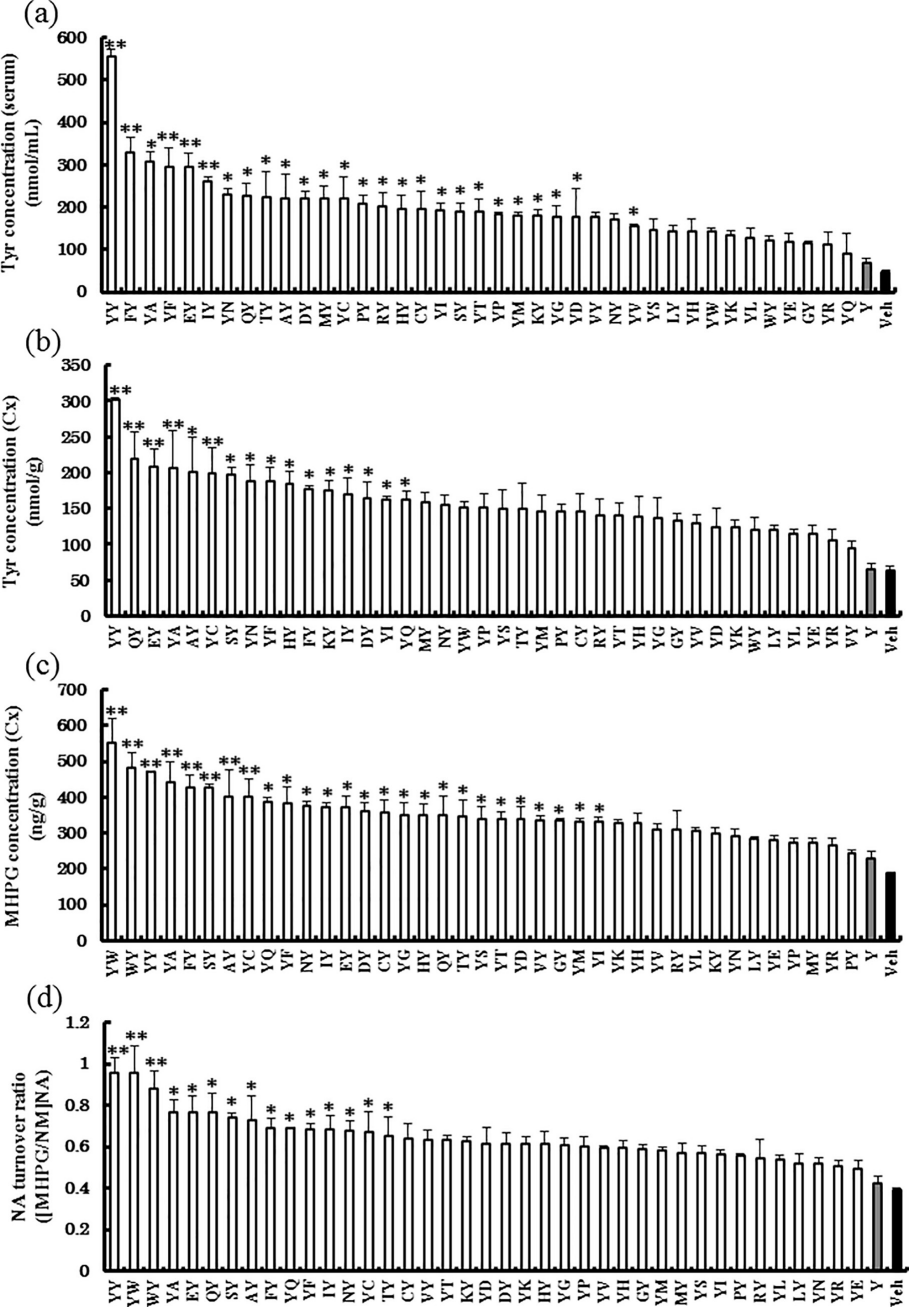
Effects of the oral administration of 39 Tyr-containing dipeptides, Tyr alone, and Vehicle on Tyr concentration in serum and cerebral cortex (Cx), and on 3-methoxy 4-hydroxyphenylglycol (MHPG) concentration in the Cx. (a) Tyr concentration in serum at 30 min after oral administration. (b) Tyr concentration in the Cx at 30 min after oral administration. (c) MHPG concentration in the Cx at 30 min after oral administration. (d) Norepinephrine turnover ratio at 30 min after oral administration. Values are means ± S.E.M. (*n* = 3). Differences between groups were analyzed using one-way analysis of variance followed by Dunnett’s test. *p < 0.05, **p < 0.001 vs. Vehicle.

Thirty-nine Tyr-containing dipeptides, Tyr alone, or physiological saline were administered, and then blood and brain Tyr concentrations and brain MHPG concentrations were measured after 30 min. The blood concentration of Tyr, which is a precursor of catecholamines, was highest for Tyr-Tyr administration (Fig 1A). Similarly, the brain concentration of Tyr was also highest for Tyr-Tyr (Fig 1B). MHPG concentration, the main metabolite of NE in the Cx, was the highest following Tyr-Trp administration (Fig 1C). NE metabolic turnover ([MHPG + NM] / NE) was high in the Tyr-Trp and Tyr-Tyr groups (Fig 1D). Tyr-Trp administration resulted in the highest values of NM and MHPG (S3 Fig). NE concentration in the Cx of the Tyr-Trp group was 1.5 times higher than that of the Tyr-Tyr group (S3 Fig). That is, Tyr-Trp seemingly increased NE synthesis and metabolism.

**Fig 2:**
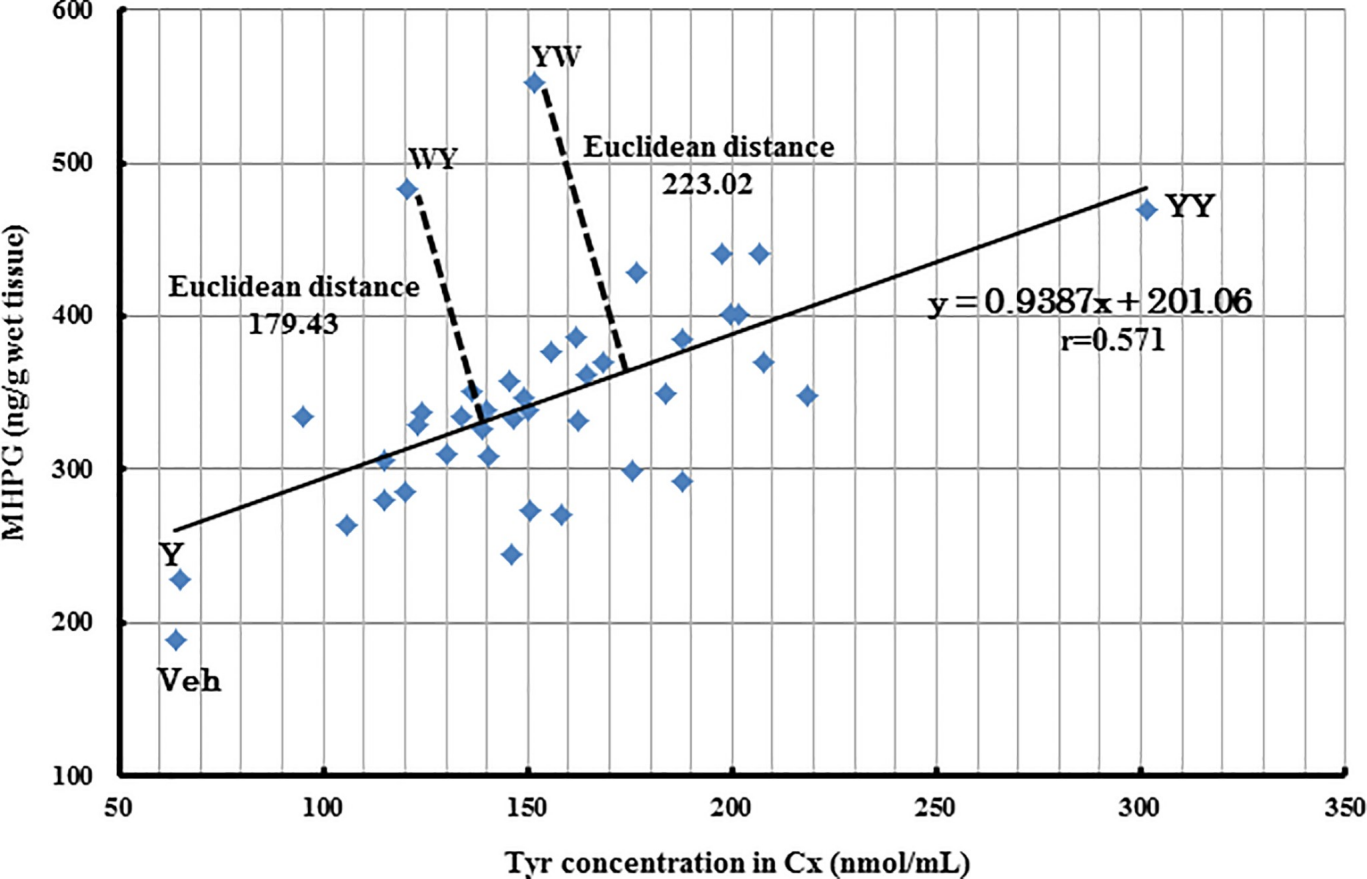
Relationship between MHPG and Tyr concentrations in the cerebral cortex (Cx).

We found a positive correlation between MHPG concentration and brain Tyr concentration for most of the dipeptides tested, including Tyr-Tyr (*y* = 0.9387*x* + 201.06, *r* = 0.571; Fig 2). However, the Tyr-Trp and Trp-Tyr groups plotted at positions away from this correlation. Tyr-Trp in particular plotted farthest from the regression line with a Euclidean distance of 223.02, while Trp-Tyr plotted a little closer with a Euclidean distance of 179.43.

### Tyr-Trp intake ameliorates the short-term memory impairment observed in AD model mice

**Fig 3:**
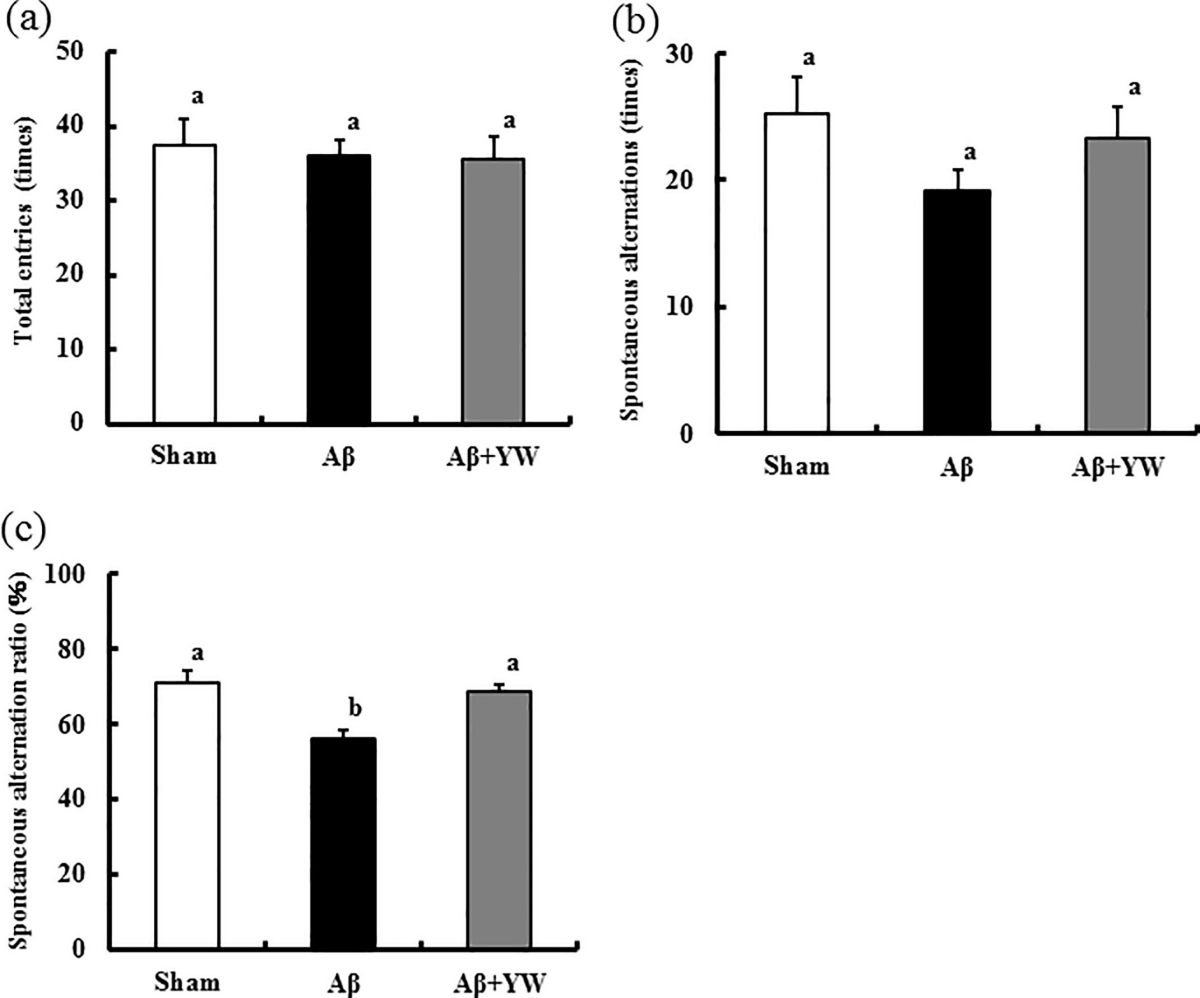
Effect of Tyr-Trp oral administration on the results of the Y-maze test. (a) Number of total entries in the Y-maze test. (b) Number of spontaneous alternations. (c) Spontaneous alternation ratio. Values are means ± S.E.M. (*n* = 10). Differences between groups were analyzed using one-way analysis of variance followed by Tukey’s honestly significant difference test. Columns with different letters indicate significant differences at p < 0.05.

Using the Y-maze test, we investigated whether Tyr-Trp ameliorates the short-term memory impairment observed in our AD model mice, which we created using intraventricular administration of Aβ peptide 25–35. There was no difference in the total number of arm entries between the groups (Fig 3A), that is, no difference was found in spontaneous behavior between the groups. In addition, there was no difference in spontaneous alternation behavior between the groups (Fig 3B). However, the spontaneous alternation rate was lower in the Aβ group than in the Sham group, indicating short-term memory impairment in these animals (Fig 3C).

Tyr-Trp administration caused a significant improvement in the short-term memory impairment of the Aβ group (Fig 3C, compare the Aβ group with the Aβ+YW group). There was no statistical difference between the Aβ+YW and Sham groups. These results indicate that the chronic oral administration of Tyr-Trp ameliorated the short-term memory dysfunction caused by Aβ peptide 25–35 (Fig 3C).

### DNA microarray analysis of mRNA expression in AD model mice

To elucidate the molecular mechanism underlying the specific action of Tyr-Trp on the brain of the AD model mice, we conducted DNA microarray analysis of mRNA expression in the Cx of the Sham, Aβ, and Aβ+YW groups. We found that Aβ treatment induced the upregulation of 281 genes and downregulation of 291 genes (false discovery rate < 0.05), when compared with the Sham group (Table 1). Among the upregulated genes, the expression of 153 genes was significantly suppressed in the Aβ+YW group compared with the Aβ group. In contrast, the expression of 179 of the downregulated genes in the Aβ group was significantly restored in the Aβ+YW group. KEGG pathway enrichment analysis demonstrated that the 153 DEGs suppressed in the Aβ+YW group showed the significant enrichment of 7 KEGG pathways (Table 1, S2 Table). The top ranked pathway was “Chemokine Signaling” (mmu04062), which includes chemokine (C-C motif) ligand 19 (*Ccl19*) and 27 (*Ccl27*) (S2 Table). In contrast, 3 KEGG pathways were significantly enriched in the 179 DEGs restored in the Aβ+YW group. The top 3 pathways of these DEGs were “Ribosome” (mmu03010), “Cocaine Addiction” (mmu05030), and “Dopamine Synapse” (mmu04728) (Table 1, S3 Table). The Cocaine Addiction and Dopamine Synapse pathways include tyrosine hydroxylase (*Th*), L-dopa decarboxylase (*Ddc*), and dopamine receptor D2 (*Drd2*), which are involved in the catecholamine biosynthetic pathway and dopaminergic transmission (S3 Table).

**Table 1:** Significant KEGG pathways detected by analyzing every differentially expressed gene (DEG) in Alzheimer’s disease model mice.

Aβ group compared with Sham group	Aβ+YW group compared with Aβ group	The number of DEGs	KEGG pathway (Benjamini-Hochberg FDR<0.05)
Upregulation 281 genes	Downregulation	153	• Chemokine signaling pathway • Retrograde endocannabinoid signaling • Nicotine Addiction • GABAergic synapse • Morphine addiction • Cytokine-cytokine receptor interaction • MAPK signaling pathway
Not Change 21634 genes	-	-	-
Downregulation 291 genes	Upregulation	179	• Ribosome • Cocaine addiction • Dopaminergic synapse
Total 22206 genes	-	Total 332	-

### Metabolome analysis reveals a distinct effect of Tyr-Trp on brain catecholamine metabolism

**Fig 4:**
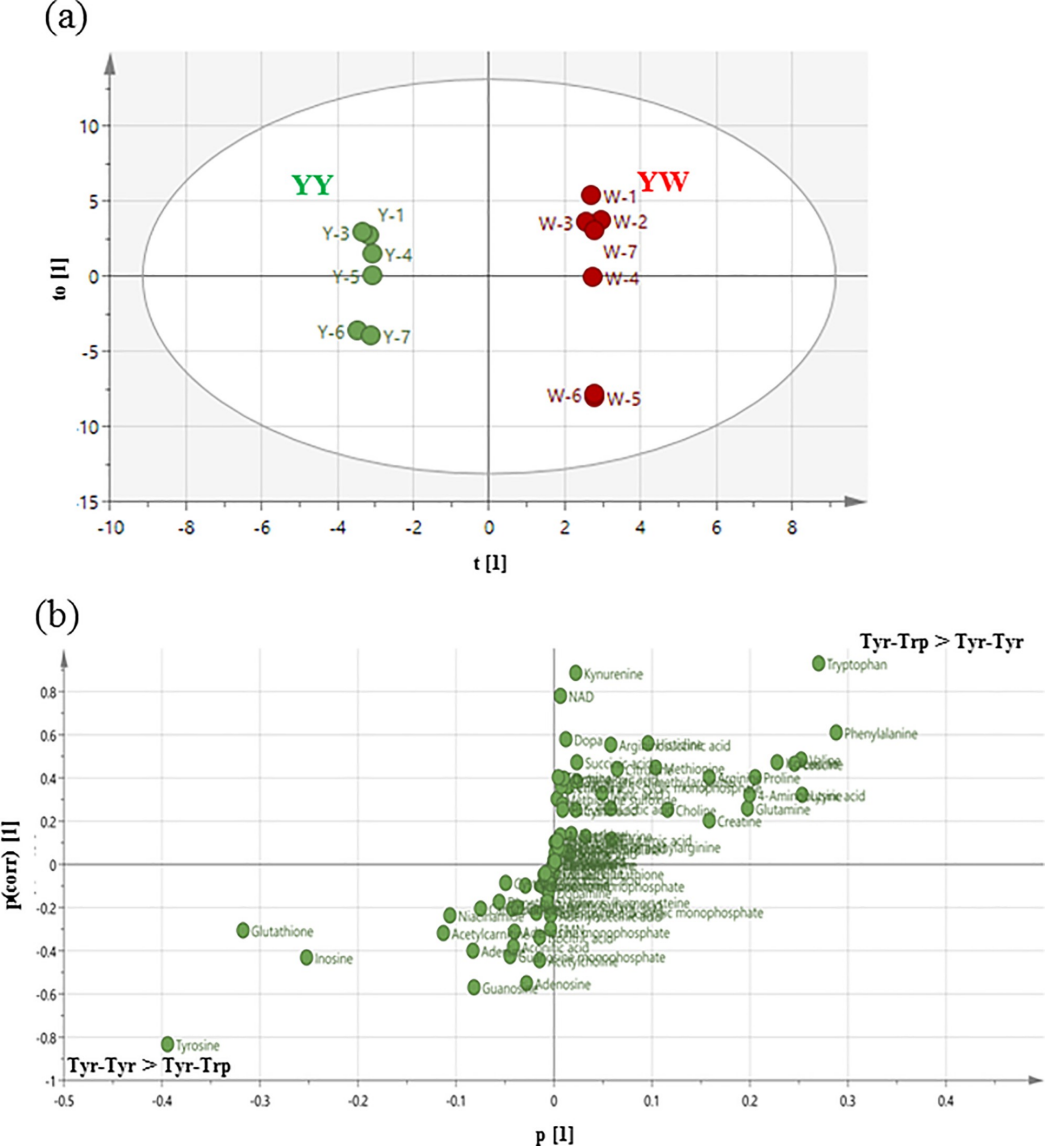
Metabolome analysis of Tyr-Trp vs. Tyr-Tyr. (a) Score plot of orthogonal projections to latent structure discriminant analysis and (b) loading S-plot of orthogonal projections to latent structure discriminant analysis derived from liquid chromatography–mass spectrometry/mass spectrometry data sets (Tyr-Trp vs. Tyr-Tyr).

The present microarray and pathway enrichment analysis suggested the involvement of catecholamine metabolism and/or its neuronal signaling in the effect of YW treatment. To elucidate the molecular mechanism underlying the effect of Tyr-Trp administration on brain metabolomes, we carried out comparative metabolomics analysis of Tyr-Trp- and Tyr-Tyr-treated Cx because this comparison could delineate which metabolomes are specific to Tyr-Trp. OPLS-DA clearly distinguished the Tyr-Tyr and Tyr-Trp groups (Fig 4A). Furthermore, a loading S-plot (Fig 4B) revealed that Trp, kynurenine, nicotinamide adenine dinucleotide (NAD), and L-dopa were among the substances that were specifically augmented by Tyr-Trp (S4 Table). Tyr was specifically augmented by Tyr-Tyr administration (S4 Table). L-dopa was not augmented by Tyr-Tyr, even though its precursor Tyr was.

## Discussion

Recent studies have identified regulatory activities of various oligopeptides generated from food proteins in functions of peripheral tissues [25,26,27]. Clinical studies have demonstrated that the peptides generated from food proteins exert a beneficial effect on the central nervous system and improves cognitive function [28,29].

We have reported that the oral administration of Ser-Tyr, a Tyr-containing dipeptide sequence frequently found in glycinin and β-conglycinin, which are the main storage proteins in soybean, may improve brain function by promoting NE synthesis and metabolism in the brain [17]. Although a comparison of Ser-Tyr, Ile-Tyr, and Tyr-Pro suggested that the non-Tyr amino acid in Tyr-containing dipeptides influences the production of MHPG in the brain, a comprehensive understanding of how Tyr-containing dipeptides affect brain NE metabolism has not been established. Thus, in this study, we compared the effect of 39 Tyr-containing dipeptides on promoting MHPG production to investigate the mechanism of exogenous Tyr-containing dipeptide action in the brain. We found that Tyr-Trp was the most potent of the Tyr-containing dipeptides in increasing brain MHPG and the NE metabolic turnover rate (Fig 1C and 1D). Surprisingly, all Tyr-containing dipeptides were more effective in increasing blood and brain Tyr levels as well as MHPG than the same quantity (mol) of Tyr alone (Fig 1B and 1C). This is the first comprehensive analysis to show that brain Tyr availability is affected by the amino acid to which Tyr is bound as a dipeptide. Ito *et al*. performed an *in vitro* evaluation of the affinity of the peptide transporter hPEPT2 (solute carrier family 15 [oligopeptide transporter], member 1 [SLC15A1]) for dipeptides. They also found a high affinity for Tyr-Trp, even though the Tyr-Trp dipeptide consists of two highly hydrophobic amino acids [30]. SLC15A1 also showed high affinity for other Tyr-containing dipeptides, and thus they are presumably well-absorbed in the intestines. These reports suggest that the higher affinity of Tyr-Trp for SLC15A1 leads to its efficient absorption in the intestines. Since oral administration of each of Tyr-containing dipeptides resulted in a significant increase in Tyr in the serum and brain (Fig 1), a certain amount of Tyr-Trp is likely to be digested to Tyr and Trp after the absorption in the intestines. Then, the resultant amino acids are transported into the brain across the blood-brain barrier probably via LAT1 and/or other amino acid transporters. After entering the brain, Tyr and Trp are likely to contribute differentially to the catecholamine metabolism and higher function.

Previous studies demonstrated that NE network dysfunction occurs in the early phase of AD etiology and is associated with declining cognitive function [6,7]. Different types of AD model mice have been created using the administration of molecules and gene recombinant technologies [31]. Among these, the learning and memory impairment model induced by the intraventricular injection of Aβ peptide is one of the most widely used AD models. Our findings showed that the short-term memory impairment observed in these AD model mice was ameliorated by Tyr-Trp intake, which was the most effective Tyr-containing dipeptide for increasing MHPG concentration and NE turnover (Fig 3C). In our protocol, the mice were given Tyr-Trp for a total of 2 weeks, that is, the week before Aβ peptide injection and the week after injection and before the Y-maze test. Thus, it is plausible that Tyr-Trp administration worked by preventing the onset of AD induced by Aβ peptide injection, suppressing AD progression, or both.

DAVID analysis of the microarray data identified enriched canonical pathways that are potentially relevant to the observed ameliorating effect of Tyr-Trp administration on the behavioral deficit of AD model mice (Tables 1, S2 and S3). The present pathway analysis demonstrated that the Chemokine Signaling pathway including *Ccl19* and *Ccl27* was highly enriched in the DEGs upregulated in the Cx of the Aβ group, but suppressed by YW in the Cx of the Aβ group. *Ccl27* has chemoattractant properties for memory T cells and is constitutively expressed in the brain [32], whereas its pathobiological functions in the brain remain largely unexplored. In contrast, *Ccl19* is upregulated in the brain of patients with multiple sclerosis and seems to contribute to the pathological maintenance of immune cells in damaged brain regions [33], although its levels are not simply associated with inflammation in these patients. Taken together, the significant enrichment of the Chemokine Signaling pathway in DEGs suppressed by YW in the Cx of the Aβ group strongly suggests that YW treatment ameliorates pathogenic immune reactions in the brain treated with Aβ peptide 25–35.

Ribosome was the top-ranked KEGG pathway enriched in the DEGs downregulated in the Cx of the Aβ group compared with the Sham group and upregulated by YW in the Aβ group. Impaired ribosomal function and the reduced expression of ribosomal components including ribosomal proteins such as RPL23A, RPL26, and RPL31 are observed in postmortem brains of AD patients [34,35]. The KEGG pathways of Cocaine Addiction and Dopamine Synapse were the 2nd and 3rd most relevant pathways enriched in the same DEGs. Both pathways contain *Th*, *Ddc*, and *Drd2*. It has been considered that dysfunction of the dopamine system has a causal relationship with the behavioral deficits seen in AD patients [36]. Indeed, the reduced expression of *TH* and *DRD2* in the postmortem brain of AD patients is consistent with this notion [37,38]. Although the mechanistic link between Tyr-Trp treatment and the increased expression of these genes remains largely unknown, these observations suggest that Try-Trp may exert a protective or preventive function against the reduced expression of ribosomal proteins and dopamine synthetic enzymes/receptors caused by the Aβ peptide.

Focusing on Trp metabolism, we identified *Qdpr*, which encodes Dhpr mRNA, as a significantly upregulated gene in the Aβ+YW group compared with the Aβ group (no significant difference between the Aβ and Sham groups, data not shown). Dhpr catalyzes the generation of BH_4_, which acts as a cofactor of Th. Thus, increased Trp metabolism by Tyr-Trp administration might enhance DA and NE synthesis via the enhanced supply of BH_4_ to Th.

**Fig 5:**
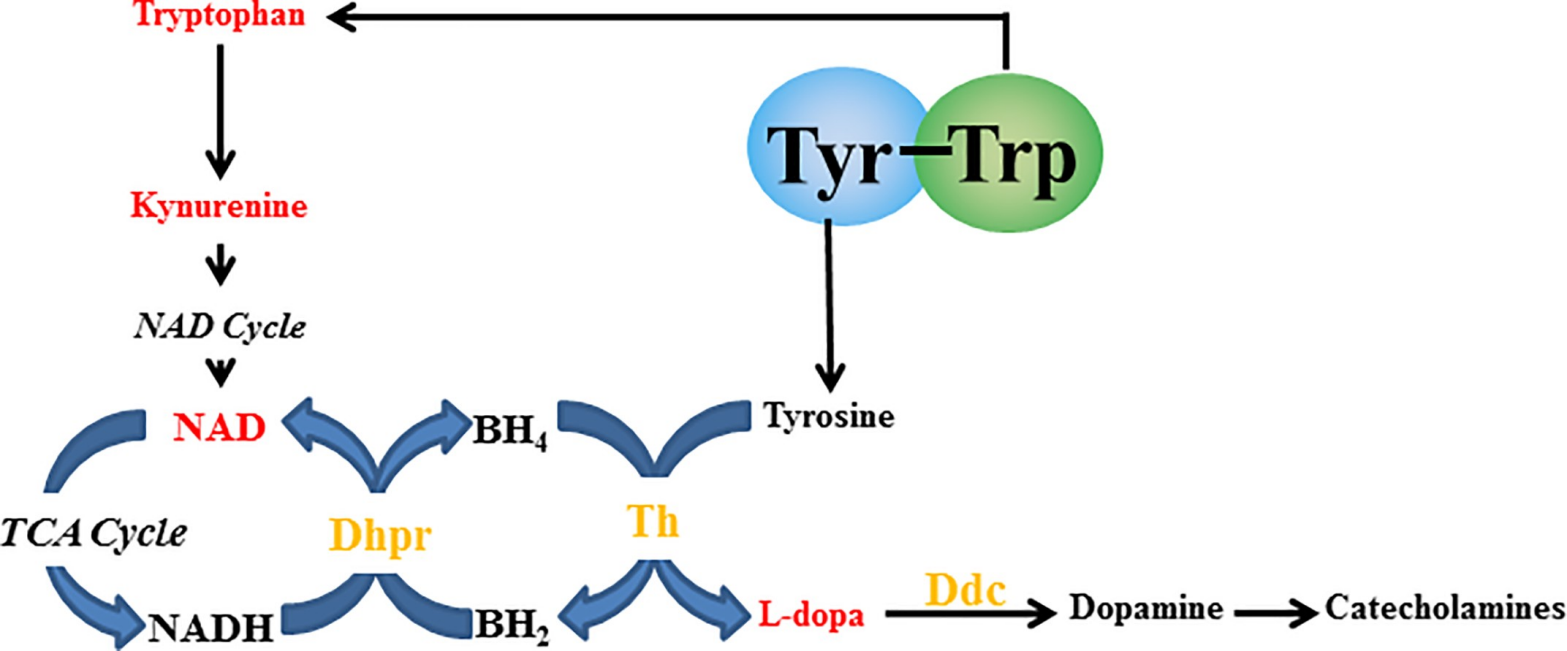
Prediction of the specific Tyr-Trp mechanism of norepinephrine synthesis and metabolism. Red indicates metabolites or materials detected by the loading S-plot in the Tyr-Trp group. Yellow indicates the enzymes detected by DNA microarray analysis. Dhpr, quinoid dihydropteridine reductase; Th, tyrosine hydroxylase; Ddc: dopa decarboxylase.

Many Tyr-containing dipeptides showed a positive correlation with MHPG and Tyr concentrations in the Cx (Fig 2). However, Tyr-Trp and Trp-Tyr were distributed away from the regression line, especially Tyr-Trp. Although Tyr-Tyr was the most effective dipeptide in increasing the concentration of Tyr in the Cx, Tyr-Trp was the most effective in increasing the concentration of MHPG in the Cx. This suggests that the increase in brain Tyr concentration is not necessarily the most important factor in increasing brain MHPG levels, but other factors are also involved. In addition, we found that Trp metabolism might affect brain NE synthesis and metabolism, which is interesting because the most characterized role for Trp in brain function thus far has been as a precursor to serotonin. Tyr-Trp might promote brain NE synthesis and metabolism via a mechanism that is linked to Trp metabolism. To verify this, we performed brain metabolome analysis to compare the effects of Tyr-Trp and Tyr-Tyr. Our results indicated that after Tyr-Trp was digested to amino acids (Tyr and Trp) in the blood, Tyr functioned as a catecholamine precursor, while Trp contributed to the supply of NAD (Fig 5), which is probably generated via the kynurenine pathway. Because Trp is also metabolized to acetyl CoA via 2-amino-3-carboxymuconic semialdehyde [39], NAD and acetyl CoA enter the citric acid cycle to produce NADH, which functions as a coenzyme for Dhpr in the metabolism of quinonoid dihydrobiopterin to BH_4_ [40]. Through this metabolic pathway, Trp administered as Tyr-Trp increases brain NAD production, which contributes to effective BH_4_ formation and consequently supplies the coenzyme to Th, which is involved in the synthesis of catecholamines by converting Tyr to L-dopa. Because the rate-limiting enzyme in the metabolism of catecholamine from Tyr is Th [41], Tyr-Trp intake presumably promoted NE synthesis and metabolism by supplying the catecholamine precursor Tyr and the Th coenzyme BH_4_ (Fig 5). Indeed, Tyr-Tyr did not increase brain MHPG as much as Tyr-Trp (Fig 1C) and scored lower than the Vehicle group for NE (S3 Fig), even though it increased the concentration of Tyr in the brain more than the other Tyr-containing dipeptides (Fig 1B). Although Tyr-Tyr effectively increased blood and brain Tyr concentrations and promoted NE metabolism, it presumably did not promote NE synthesis because of the shortage of Th coenzyme BH_4_. Tyr-Trp intake led to high concentrations of L-dopa, MHPG, and NM (Figs 4B and 1C and S3 Fig) as well as higher NE concentration compared with the Vehicle group (S3 Fig). Presumably, Tyr-Trp administration facilitated NE synthesis and metabolism by simultaneously providing Tyr and BH_4_ via Trp metabolism, as predicted by metabolome analysis. Metabolome analysis of postmortem brains has indicated abnormal Tyr metabolism in AD patients [42,43]. From the aforementioned pathway, Tyr-Trp administration presumably inhibits the onset and progression of AD by supplying the coenzyme to Th, the rate-limiting enzyme of Tyr metabolism. Details of the Tyr metabolism-regulating effect of Tyr-Trp should become clearer by observing the effect of Tyr-Trp administration on Th activity.

This study demonstrated that among the Tyr-containing dipeptides, Tyr-Trp promoted NE synthesis and metabolism the most. Chronic Tyr-Trp administration ameliorated the short-term memory impairment of AD model mice. The present DNA microarray and metabolome analyses suggest that Tyr-Trp facilitated NE synthesis and metabolism through Tyr and Trp metabolism. We used only one type of AD mouse model for validation, so the full extent of the effect of Tyr-Trp and its detailed mechanism of action should be elucidated by performing additional validations with transgenic AD mouse models that develop the disease spontaneously as well as other mouse models of dementia. In conclusion, Tyr-Trp specifically enhanced brain NE metabolism and alleviated the memory impairment caused by Aβ peptide in an animal model.

## Supporting information

S1 Fig qFARMS principal component analysis with all sample data. Red indicates the Sham group. Black indicates the Aβ group. Blue indicates the Aβ+YW group.
(TIF)

S2 Fig qFARMS principal component analysis without G1.2 data of the Sham group. Red indicates the Sham group. Black indicates the Aβ group. Blue indicates the Aβ+YW group.
(TIF)

S3 Fig Effects of the oral administration of 39 Tyr-containing dipeptides, Tyr, and Vehicle on norepinephrine (NE) and normetanephrine (NM) concentrations in the cerebral cortex (Cx). (a) NE concentration in the Cx at 30 min after oral administration. (b) NM concentration in the Cx at 30 min after oral administration. Values are means ± S.E.M. (*n* = 3). Differences between groups were analyzed with one-way analysis of variance followed by Dunnett’s test. There were no significant differences vs. Vehicle.
(TIF)

S1 Table Complete optimized target list. (TIF)

S2 Table Significant KEGG pathways detected by DEGs that were upregulated in the Aβ group compared with the Sham group and downregulated in the Aβ+YW group compared with the Aβ group. (TIF)

S3 Table Significant KEGG pathways detected by DEGs that were downregulated in the Aβ group compared with the Sham group and upregulated in the Aβ+YW group compared with the Aβ group. (TIF)

S4 Table p(corr) absolute values of the loading S-plot. (TIF)

S1 File DEGs detected by DNA microarray. (XLSX)

S2 File Metabolome analysis data. (XLSX)
