# Supplementary figures and images for: Tyr-Trp administration facilitates brain norepinephrine metabolism and ameliorates a short-term memory deficit in a mouse model of Alzheimer’s disease
Source: PLoS One. 2020 May 4;15(5):e0232233. doi: 10.1371/journal.pone.0232233 (PMC7197849; doi:10.1371/journal.pone.0232233)

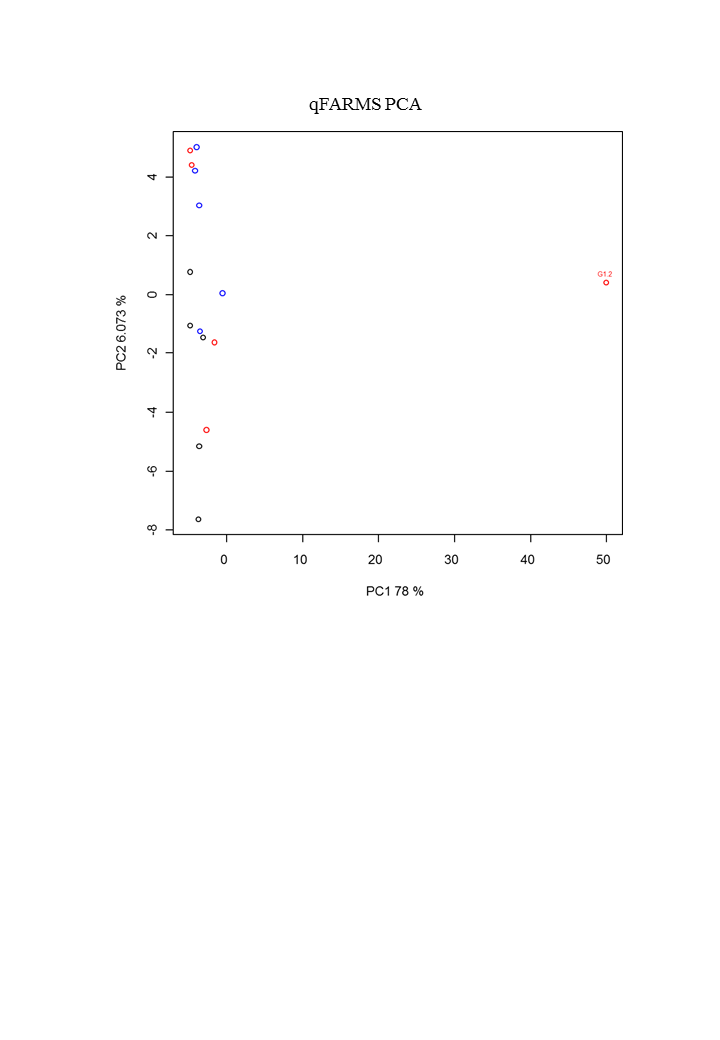

Supplement: S1 Fig — Red indicates the Sham group. Black indicates the Aβ group. Blue indicates the Aβ+YW group. (TIF) [file pone.0232233.s001.tif]

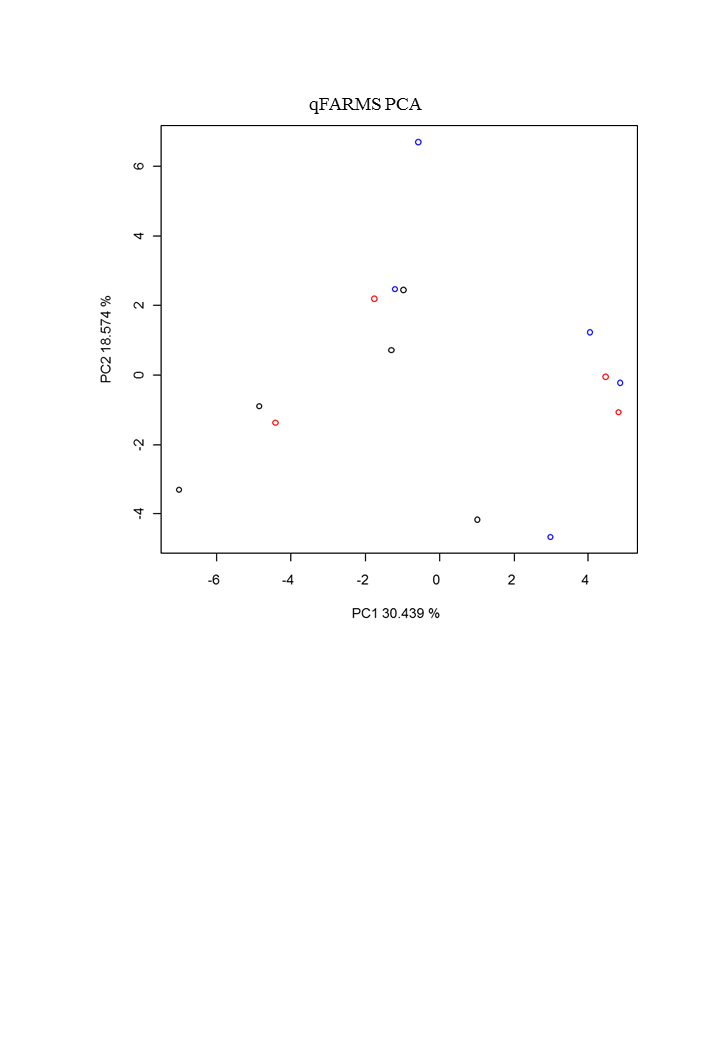

Supplement: S2 Fig — Red indicates the Sham group. Black indicates the Aβ group. Blue indicates the Aβ+YW group. (TIF) [file pone.0232233.s002.tif]

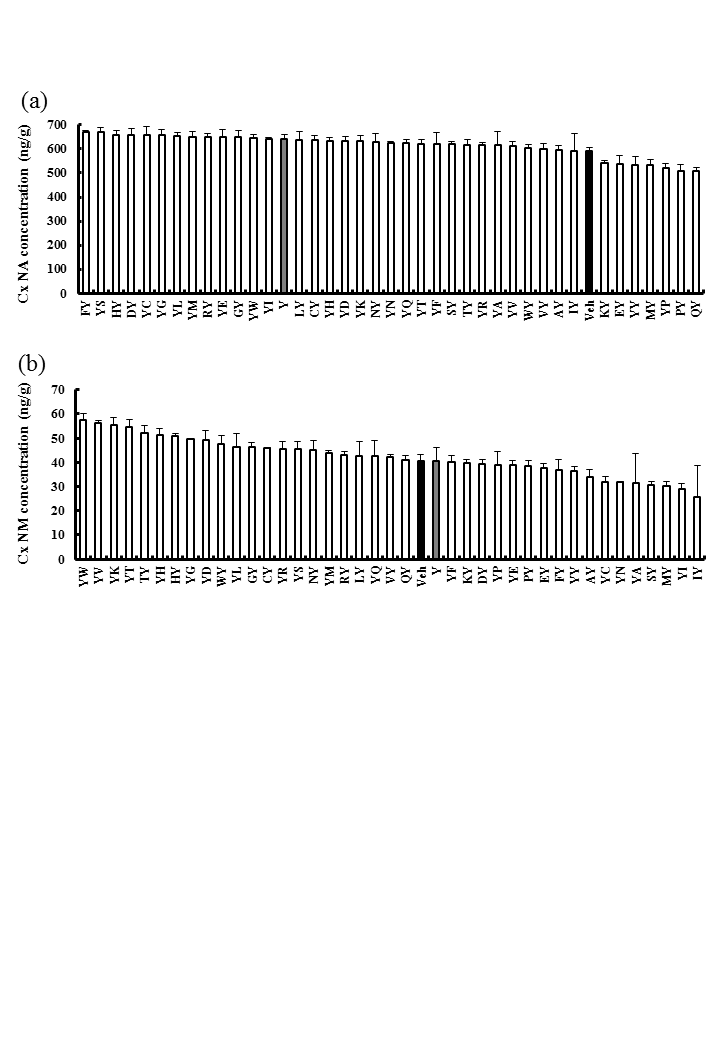

Supplement: S3 Fig — (a) NE concentration in the Cx at 30 min after oral administration. (b) NM concentration in the Cx at 30 min after oral administration. Values are means ± S.E.M. (n = 3). Differences between groups were analyzed with one-way analysis of variance followed by Dunnett’s test. There were no significant differences vs. Vehicle. (TIF) [file pone.0232233.s003.tif]

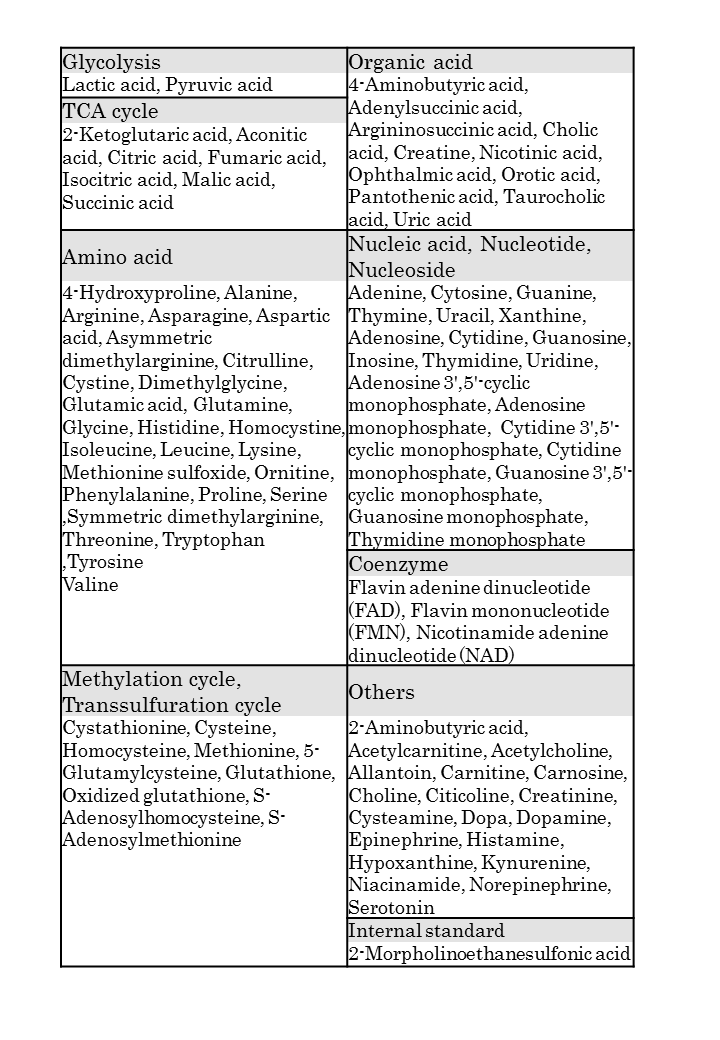

Supplement: S1 Table — (TIF) [file pone.0232233.s004.tif]

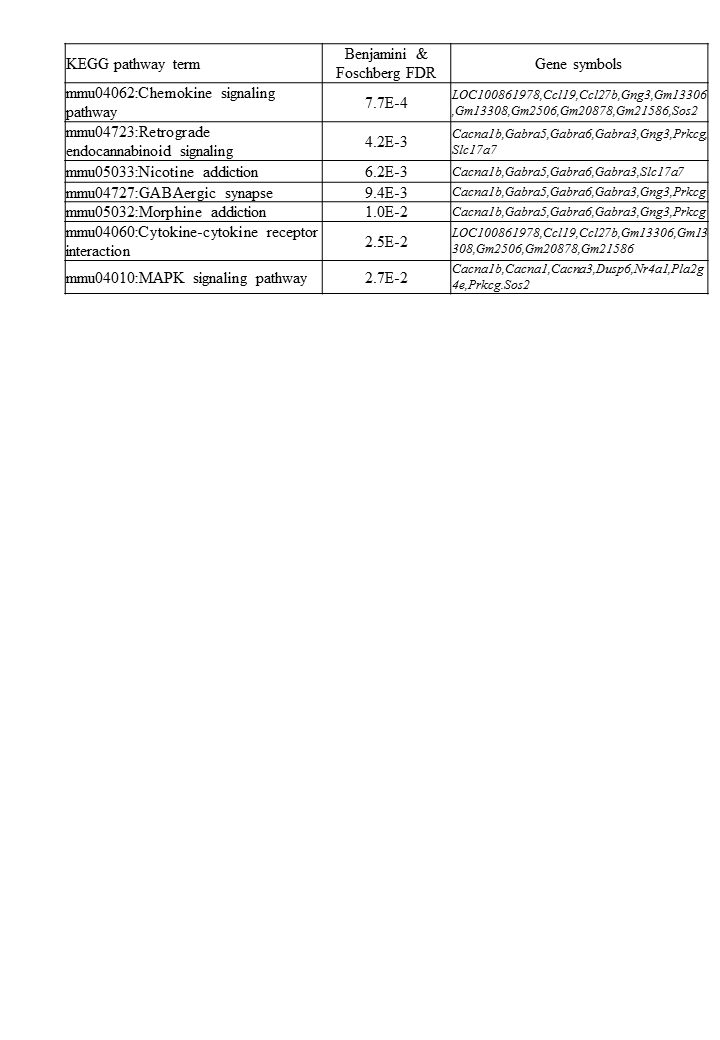

Supplement: S2 Table — (TIF) [file pone.0232233.s005.tif]

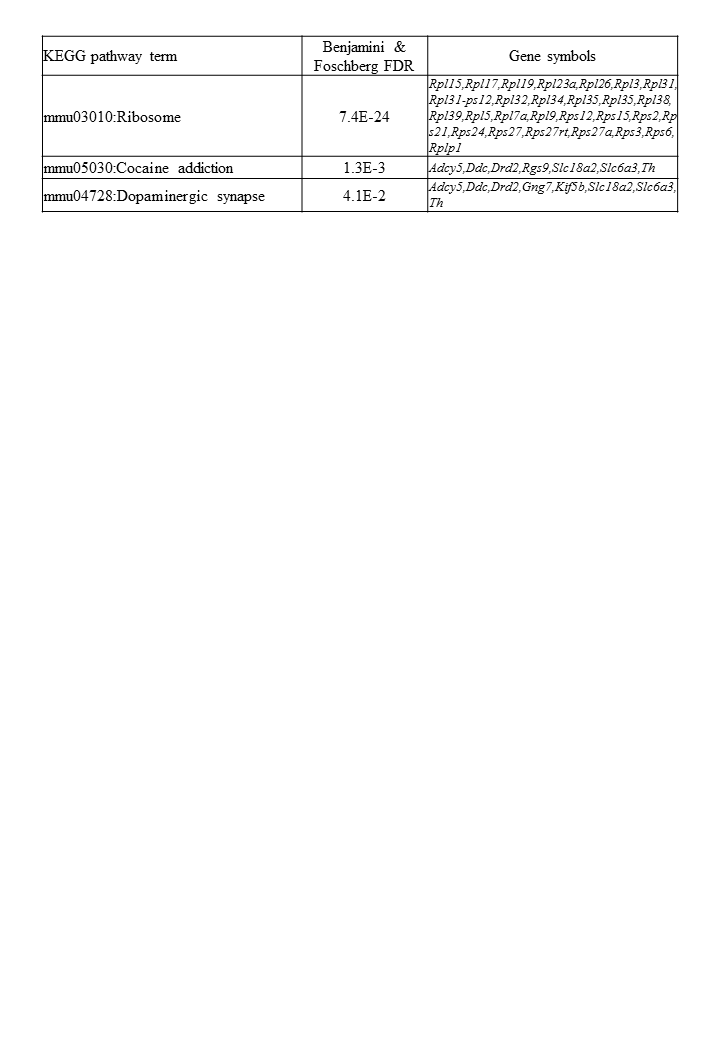

Supplement: S3 Table — (TIF) [file pone.0232233.s006.tif]

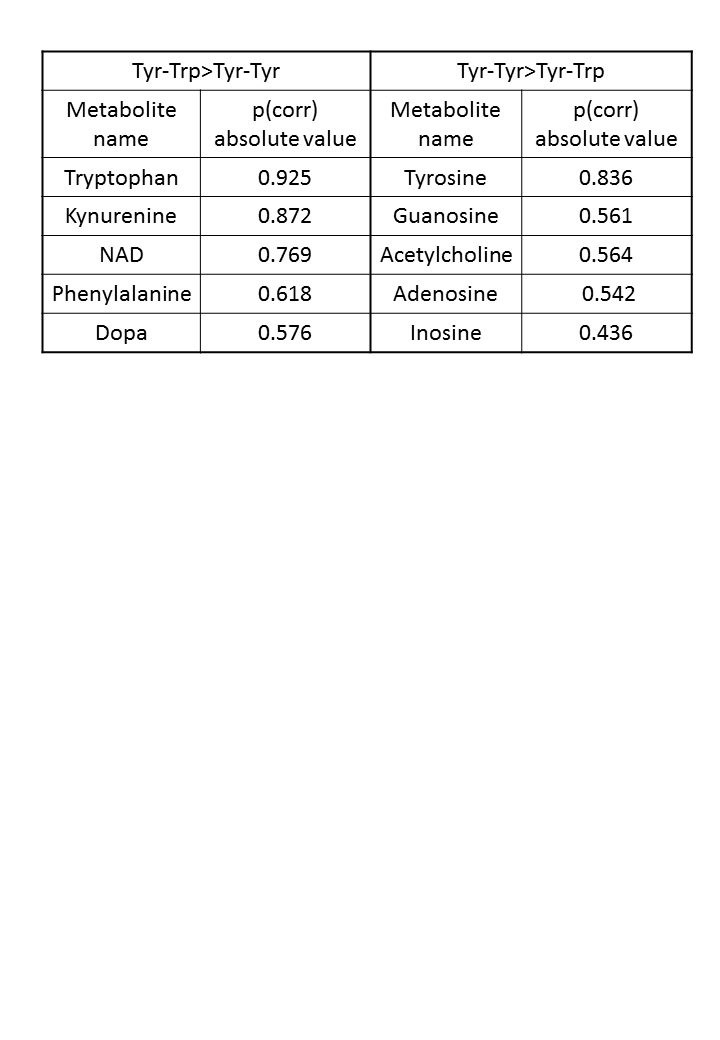

Supplement: S4 Table — (TIF) [file pone.0232233.s007.tif]
